# Experimental and Theoretical Studies on Hysteretic Behavior of Friction Energy Dissipation Composite Chord under Quasi-Static Tests

**DOI:** 10.3390/ma16072885

**Published:** 2023-04-04

**Authors:** Qishi Zhou, Wenwu He, Zhibin Zhou, Wenxuan Guo, Shuaishuai Liu

**Affiliations:** School of Civil Engineering, Central South University, Changsha 410075, China

**Keywords:** staggered truss, friction energy dissipation, composite chord, seismic performance, hysteretic test

## Abstract

To improve the seismic performance of a staggered truss steel framing system, the basic force unit in the truss system is replaced by a friction energy dissipation truss. The difference between a friction energy dissipation truss and an ordinary truss is that the upper chord is a friction energy dissipation composite chord. In this paper, we investigate the effects of the number of bolts and the friction surface on the energy dissipation capacity of the chord by a quasi-static test on six composite chord specimens at a scale of 1:2. The results show that the hysteresis curves of friction energy dissipation composite chords are ideal rectangles, and the energy dissipation capacity is excellent. The more bolts there are in the specimen, the slower the energy dissipation capacity of the chord decreases. Among the different friction surface specimens, the energy dissipation capacity of the aluminum friction plate specimen decays the fastest, while the energy dissipation capacity of the shot-blasted treated specimen decays substantially after the first cycle. Friction plates can improve the stability of the hysteresis properties. Based on the test results, this paper proposes a calculation method for the ultimate bearing capacity of the composite chord, which provides a basis for the design of a friction energy dissipation truss. In addition, we studied the effects of different bolt clamping forces and slotted bolt hole lengths on the energy dissipation capacity of composite chords by establishing a finite element analysis. It was shown that as the clamping force of the bolt increases, the energy dissipation capacity of the specimen becomes stronger but the stability decreases. The energy dissipation capacity of the chord is close to a linear relationship with the slotted bolt hole lengths; thus, increasing the slotted bolt hole lengths within the allowable range of inter-story drifts can enhance the energy dissipation capacity of the chord. Finally, we propose the design method of the angle steel by analyzing the force of the chord.

## 1. Introduction

**Figure 1:**
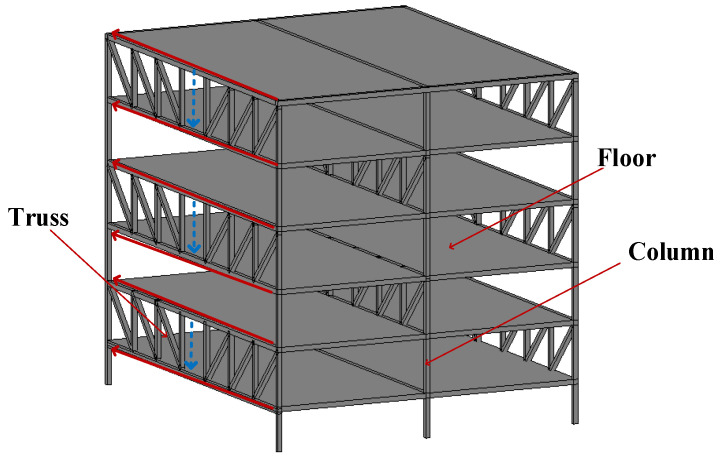
Horizontal force transmission path of a staggered truss steel framing system.

A staggered truss steel framing system is an economical, green, and flexible structural system that can provide larger space without columns. The main components can realize full prefabricated assembly construction, and the steel can be recycled, which is in line with the development trend of green building [1,2,3,4]. Figure 1 depicts the horizontal force transmission path of a staggered truss steel framing system. Through the floor, the horizontal force of the upper truss is transmitted to the upper chord of the adjacent truss, and then the force is transmitted to the lower chord through the web member. A friction damper can consume energy through sliding friction to reduce the damage caused by earthquakes. Qiu et al. [5] reported a cyclic loading test on a novel self-centering brace, which is a serial combination of shape memory alloy slip friction dampers and steel tubes. The results showed that the brace exhibits a flag-shaped hysteresis, characterized by an excellent self-centering capability and satisfactory damping capacity. Wang et al. [6] studied the hysteretic damping and self-centering characteristics of a buckling-plate self-centering friction damper (BPSCFD) by theoretical derivation and finite element simulation. The results indicate that the BPSCFD exhibits flag-shaped hysteresis and low post-yielding stiffness, and the dampers can effectively reduce the peak and residual deformation of the bridge bend without increasing peak acceleration and base shear. Qin et al. [7] investigated the coefficient of friction of the friction dampers with friction pads made of four materials under cyclic loading. The results supply a guideline for the design of a friction damper. Veismoradi et al. [8] introduced a Self-Centering Rotational Friction damper (SC-RF damper), which can provide energy dissipation and self-centering characteristics. The SC-RF dampers provide remarkable flexibility between the connection component design and structural system performance. Ghorbani et al. [9] investigated the performance of a friction damper with two slip loads in controlling the seismic response of steel moment-resisting building structures. The results show that this damper effectively reduces the seismic response of the structure in terms of story drifts, residual drifts, absolute floor accelerations, and base shear forces. At present, numerous theoretical analyses and experimental studies have shown that the performance of a friction damper is stable, the energy dissipation capacity is good, and that a friction damper can significantly improve the seismic performance of a structure [10,11,12,13,14,15,16].

However, in a high earthquake intensity area, an ordinary staggered truss steel framing system may face the problem of insufficient ductility and energy dissipation capacity [17]. Increasing the Vierendeel panel width and vertical truss spacing ratio of a staggered truss framing system can improve the ductility of the structure [18]. Kim J et al. [19,20] evaluated the seismic performances of staggered truss system structures with and without Vierendeel panels, and the results showed that the use of end bracing and vertical cables was somewhat effective in enhancing strength and ductility and decreasing inter-story drifts. In addition, Kim J et al. [21] developed a design procedure for staggered truss frames with friction dampers in the Vierendeel panel and evaluated their seismic performance. The results showed that the substitution of rotational friction dampers at the location of the plastic hinges can enhance ductility and reduce failure probability. Zhou et al. [22] indicated that with an increase in the open-web panel length of the truss, the top lateral displacement and ductility of the structure increase. Mistry et al. [23] studied staggered truss systems with and without a shear wall by using ETAB software. The results showed that the staggered truss system with a shear wall is more efficient. It is not effective to enhance or improve the seismic performance of an ordinary staggered truss steel frame structure to resist earthquakes. A traditional friction damper can improve the ductility of the structure, but the energy dissipation capacity and economy are not good enough.

**Figure 2:**
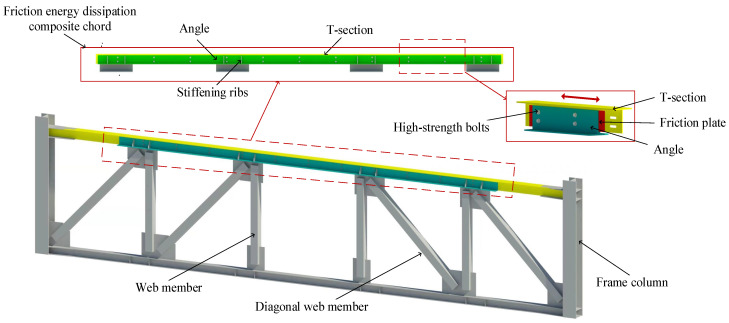
The structure diagram of the friction energy dissipation composite truss.

To obtain better ductility and energy dissipation capacity, this paper proposes converting an ordinary truss into a friction energy dissipation truss to form a new structural system: the friction energy dissipation staggered truss steel frame structure system. The difference between a friction energy dissipation truss and an ordinary truss is that the upper chord is a friction energy dissipation composite chord. The friction energy dissipation composite chord consists of T-section steel, angle steel, and friction plates connected by bolts, as shown in Figure 2. The T-section steel is attached to the column and floor, and the slotted bolt holes are set so that relative sliding can occur between it and the angle steel. The angle steel is connected to a web member, and its ends are at a certain distance from the column to ensure sufficient sliding distance. When the horizontal force of the friction energy dissipation composite chord reaches its sliding force, the friction will work to dissipate energy, thereby improving the energy dissipation capacity of the truss. Compared with an ordinary friction energy dissipation structure, the friction energy dissipation composite chord combines the friction energy dissipation in the structure, which is simple and shows better ductility and energy dissipation capacity. This paper studied the effects of the number of bolts, the friction surface, the clamping forces of the bolts, and the slotted hole lengths on the energy dissipation capacity through experimental and numerical simulations. The calculation method for the ultimate bearing capacity of the composite chord is obtained, which provides a basis for the design of the friction energy dissipation truss. Finally, we propose a design method for the angle steel by analyzing the force of the composite chord, which provides a relevant reference for the design of the friction energy dissipation composite chord and facilitates the popularization and application of the friction energy dissipation truss in a high earthquake intensity area.

## 2. Experiment Process

### 2.1. Specimens

**Figure 3:**
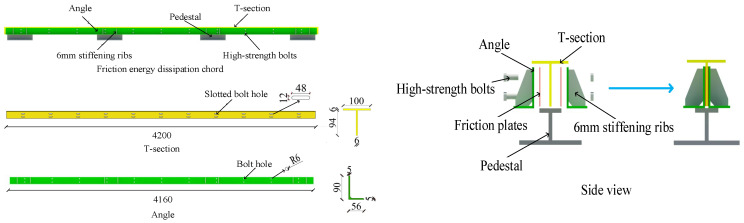
The structure diagram of the friction energy dissipation composite chord specimen (based on SJ1).

According to the specifications [24], 1:2 scaled friction energy dissipation composite chord specimen SJ1 is designed with a steel grade of Q345. As shown in Figure 3, there are several 48 mm slotted bolt holes in the T-section steel to meet the sliding displacement requirement between the T-section steel and the angle steel. The 6 mm thick stiffeners are set on one side of the angle steel, and there are bolt holes with a radius of 6 mm in the angle steel. Two angle steels are connected to the T-section steel by 10.9 grade M12 high-strength bolts, the friction plate between the angle steel and T-section is 1 mm thick, and the length is the same as the angle steel. As a pedestal, H-section steels are welded at the connection area between the chord and the truss web member, which are used to connect with the laboratory base to provide vertical and horizontal reaction force for specimens and simulate the constraint effect of a web member on the friction energy dissipation composite chord.

Based on SJ1, six friction energy dissipation composite chord specimens with a scale of 1:2 were made by changing the parameters; the details of the specimens are listed in Table 1. In order to make the friction evenly distributed, we arranged the bolts to be equally spaced.

### 2.2. Test Setup and Instrumentation

**Figure 4:**
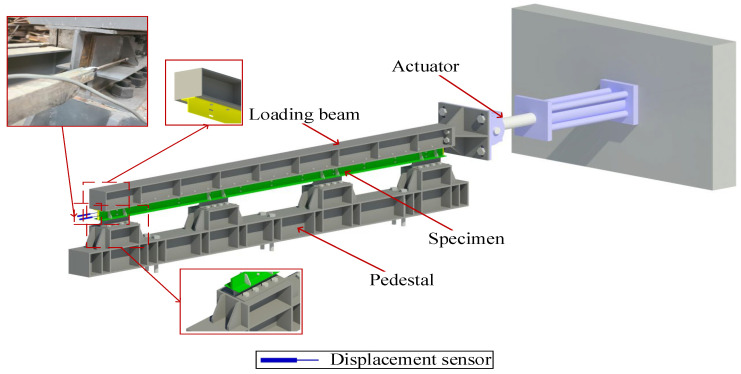
The loading device diagram of the friction energy dissipation composite chord.

The Mechanical Testing and Simulation (MTS) structure test loading system with a 100 t level actuator is used in the test. The maximum loading displacement is ±25 mm, according to the specifications [25]. The test setup is designed as shown in Figure 4. The steel base is anchored to the ground by M40 anchors and is bolted to the pedestal of the specimen. Specimens are connected to the H-section steel (250 mm × 250 mm × 9 mm × 14 mm) by bolts. The end of the H-section steel is connected with the force transducer as a loading beam to apply a horizontal load, and its longitudinal stiffness is close to the floor, which is used to simulate the effect of the floor and studs on the T-section steel. As shown in Figure 4, KTR11 linear displacement sensors with a range of ±50 mm, manufactured by MIRAN Technology, are arranged at the ends of the T-section steel and angle steel to measure their horizontal displacement. The load is measured by the built-in sensor of the servo machine, manufactured by MTS, which allows the force-displacement curve to be obtained during loading.

### 2.3. Loading Scheme

**Figure 5:**
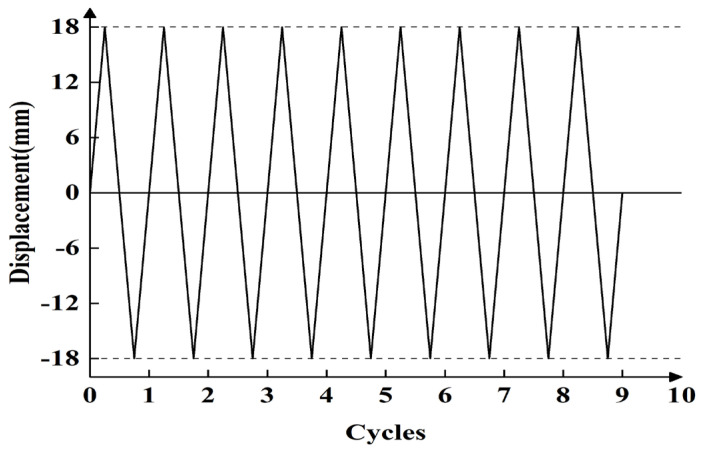
The test loading protocol of the friction energy dissipation composite chord.

Using the Coulomb law of friction to estimate the sliding friction of the specimens, a sliding load of 25% of the specimen was pre-loaded before formal loading to eliminate the effect of inelastic deformation on the test. During loading, the thrust force is applied. First, it is specified that thrust force is positive and tensile force is negative. Figure 5 depicts the loading protocol. Specimens are loaded by load control before sliding. After continuous loading to relative sliding between T-section steel and angle steel, the displacement control loading is adopted, the sliding friction force remains mostly unchanged, and the displacement is gradually increased until it is loaded to the maximum value of the sliding displacement of 18 mm. It is then loaded back to a maximum negative value of −18 mm, and continuously loaded in this way until there is no obvious change in sliding friction. The loading speed is 0.05 Hz.

## 3. Experimental Results and Discussion

### 3.1. Experimental Observation

**Figure 6:**
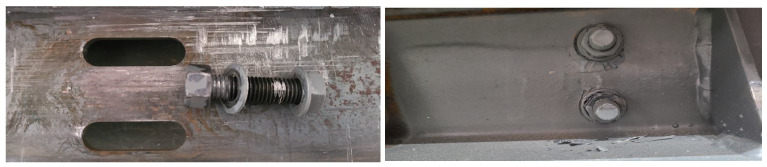
The test phenomenon of the friction energy dissipation composite chord specimens.

When specimens with different numbers of bolts were loaded, due to the mutual running-in between the friction surfaces, a discontinuous crunching sound was emitted, which gradually decreased as the number of cycles increased. Figure 6 shows the test phenomenon. The contact part between the bolts and the angle steel was peeled off due to the high stress during the test. The specimens were disassembled after testing and showed various degrees of extrusion damage on the edges of the screws and slotted bolt holes, with damage and deformation decreasing with the number of bolts and apparent friction scratches on the surface of the brass friction plates.

**Figure 7:**
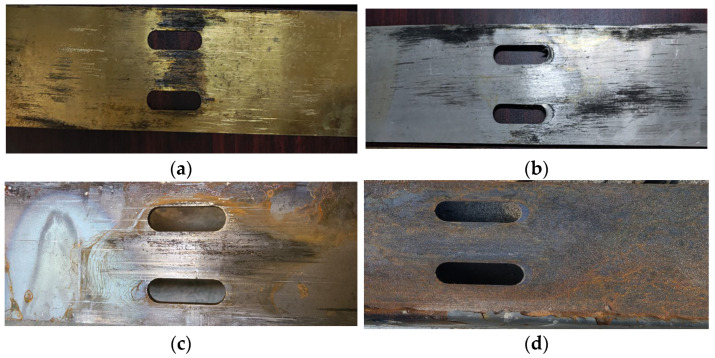
Scratches of different friction surfaces: (**a**) SJ1, brass; (**b**) SJ4, aluminum; (**c**) SJ5, no friction plates; (**d**) SJ6, shot blasting.

When specimens with different friction surfaces were loaded, the sound of SJ5 was louder and not continuous compared with SJ1 and SJ6. The sound of SJ4 with an aluminum friction plate was quieter and more continuous than SJ1. Because of the different friction materials, the wear rate and friction mechanism of friction plates are continuously changing, resulting in different sounds. As shown in Figure 7, the surfaces of the aluminum and brass friction plates show visible scratches. However, there were no deep friction scratches on the shot blasting and frictionless plates. In addition to the friction, factors affecting slip displacement include the interlocking between the frictional surfaces, which essentially provides no increase in friction when the test is loaded at a later stage. 

### 3.2. Experiment Results and Analyses

#### 3.2.1. Experiment Data

The maximum static friction force received by the specimens at the beginning of sliding is called the maximum static sliding friction force, and the force required to maintain its sliding is the sliding friction force. These are important parameters, reflecting the performance stability of the friction energy dissipation chords and a significant basis for structural stability design. In order to verify whether the sliding friction force of the specimens is stable under reciprocating load, the friction non-uniformity coefficient α is proposed according to references [26,27]:

(1) $$\alpha =\frac{\left|F_{+}\right|}{\left|F_{-}\right|}$$

where $F_{+}$ and $F_{-}$ are the average sliding friction forces under the action of thrust force and tensile force, respectively, and α reflects the imbalance of the sliding friction force. The closer α is to 1, the better the symmetry is.

The relationship between the maximum static sliding friction force and the sliding friction force can reflect the stability of the specimens during the conversion of static and dynamic friction force. Here, β is defined as the influence coefficient of the maximum static friction force according to reference [26]:

(2) $$\beta =\frac{\left|F_{0+}\right|+\left|F_{0-}\right|}{\left|F_{+}\right|+\left|F_{-}\right|}$$

where $F_{0+}$ and $F_{0-}$ are the maximum static sliding friction forces under the action of thrust force and tensile force, respectively, measured by the built-in sensor of the servo machine. The closer β is to 1, the more stable the specimen is in the static and dynamic conversion. The results are shown in Table 2.

In order to make the friction changes under different conditions comparable, the coefficient of variation C.V is introduced to judge the dispersion degree of friction:

(3) $$C.V=\frac{\sigma }{F}$$

The calculation results for C.V are shown in Table 3, where ${\bar{F}}_{\max}$ and ${\bar{F}}_{\min}$ are the maximum and minimum values of the average sliding friction in a single cycle, respectively. F is the average sliding friction, and σ is the standard deviation. The smaller the C.V, the smaller the dispersion degree of friction.

#### 3.2.2. Comparative Analysis of Different Numbers of Bolts

**Figure 8:**
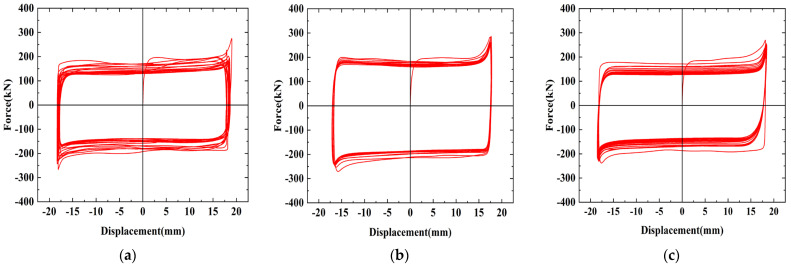
The hysteresis curves of different numbers of bolts: (**a**) SJ1, with 22 bolts; (**b**) SJ2, with 30 bolts; (**c**) SJ3, with 16 bolts.

The hysteresis curves of the friction energy dissipation composite chord specimens are shown in Figure 8 for different numbers of bolts. The initial sliding friction of the specimens is mostly the same. Brass is soft and the surface is prone to wear, resulting in a discontinuous crunching sound and uneven changes in the friction coefficient, which leads to fluctuations in the hysteresis curves. As the loading progresses, however, the rough peaks between the friction surfaces flatten out, and the curve gradually tends to smooth out. The bolt is squeezed within the slotted bolt holes, resulting in the sharp part of the curve. As loading progresses, the friction surfaces gradually become smoother after continuous cyclic wear, resulting in reduced friction. The wear tends to be stable, and the sliding friction becomes stable. In general, the hysteresis curves of the specimens are rectangular, and the hysteretic performance is good.

As can be seen from Table 2, the non-uniformity coefficient α and the maximum static friction influence coefficient β of the specimens are close to 1, the symmetry of the hysteresis curves is good, and the static and dynamic friction force conversion is relatively stable. From Table 3, it can be seen that with an increase in the number of bolts, ${\bar{F}}_{\max}/{\bar{F}}_{\min}$ and C.V become smaller, which means the smaller the sliding friction force changes with the cycle and the lower the dispersion degree of the sliding friction force. 

**Figure 9:**
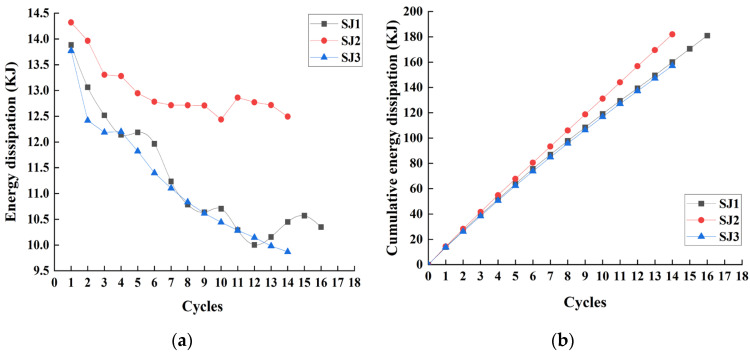
The energy dissipation analysis of SJ1, SJ2, and SJ3: (**a**) Energy dissipation; (**b**) Cumulative energy dissipation.

The envelope area of the hysteresis curves of specimens can be used to characterize the energy dissipation value of the specimens during the cycle and thus to evaluate the energy dissipation capacity of the friction energy dissipation chords. The energy dissipation of the specimens and their cumulative energy dissipation through the loading process is shown in Figure 9 for different numbers of bolts. As the number of bolts increases, the energy dissipation capacity of the specimen decreases more slowly and the cumulative energy dissipation is larger. Because the total clamping force is constant, with the increase in bolts the area of the clamping forces will be larger, the friction will be more evenly distributed, the specimen is less affected by interlocking, and the degree of wear is smaller, which makes the change in friction smaller and the attenuation of the energy dissipation capacity slower. If the bolt spacings are very close, the effective friction surfaces will overlap, resulting in smaller sliding friction or shear failure of the steel, and excessive spacing may lead to local buckling of the plate. As the results show, the energy dissipation capacity of SJ2 with 30 bolts is the slowest and the energy dissipation capacity is relatively better, the bolt spacing is 280 mm, which is about 23 times the bolt aperture $d_{0}$, and the minimum allowable distance of bolt spacing is 3$d_{0}$ [24]; therefore, it is suggested that the bolt spacing can be considered between 3~23 times the bolt aperture to design the number of bolts.

#### 3.2.3. Comparative Analysis of Different Arrangements of Friction Surfaces

**Figure 10:**
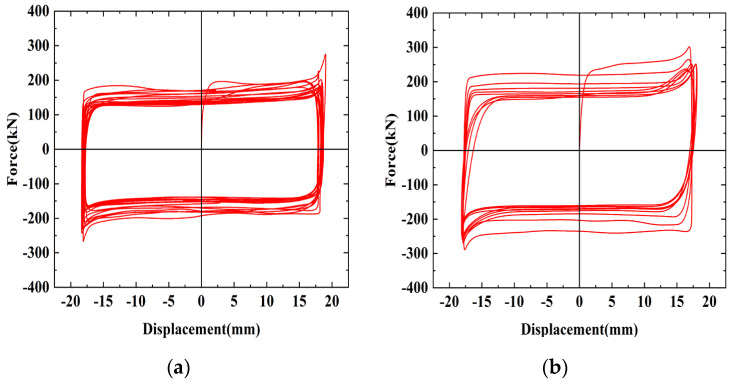

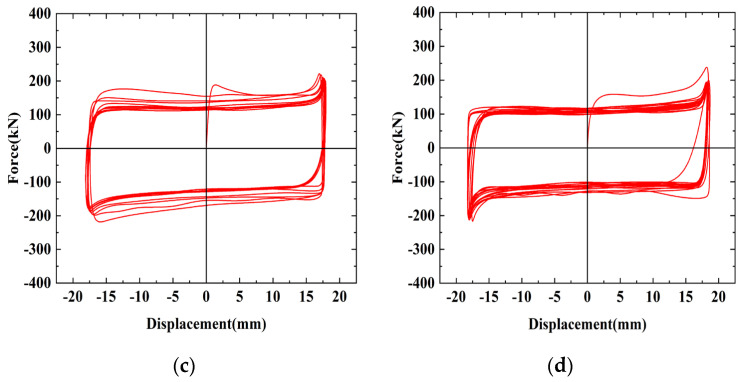
The hysteresis curves of different friction surfaces: (**a**) SJ1, brass; (**b**) SJ4, aluminum; (**c**) SJ5, no friction plates; (**d**) SJ6, shot blasting.

Figure 10 shows the hysteresis curves of the specimens with different friction surfaces. The hysteresis curve of SJ4 is relatively smooth, but the friction decreases quickly because aluminum is softer than brass and the friction surface wears faster. When SJ4 was initially loaded, the friction surface was affected by interlocking, resulting in fluctuations in the sliding load. However, the rough part of aluminum can be smoothed quickly, interlocking can be alleviated faster, and the hysteresis curves will smooth out rapidly. Due to the large initial friction coefficient of aluminum, the initial sliding load of SJ4 is the largest. The energy dissipation performance of SJ5 is not as stable as the brass and aluminum friction plates, and therefore brass and aluminum friction plates play a significant role in controlling the stability of sliding friction. The friction of SJ6 suddenly drops sharply during the second cyclic loading, which is about 18.1% lower than the first loading, because the initial sliding friction force of SJ6 is greatly affected by the tiny concave and convex surfaces caused by shot blasting, resulting in a large sliding load required for initial loading. During subsequent loading, the rough peaks on the surface are sheared due to the horizontal shear force, resulting in tiny pieces of debris falling off the steel surface between the contact surfaces. Under the influence of debris, the friction property of the specimen is between the sliding friction and the rolling friction; thus, the force value is reduced, the steel debris is gradually embedded into the steel plate during the continuous friction process and then forms a groove through repeated sliding, and the frictional resistance generated gradually increases until it is stable.

As can be seen from Table 2, the symmetry of the hysteresis curves is good, and the specimens with the friction plates are relatively smaller, indicating that the setting of a friction plate can improve the stability of the specimens during the static and dynamic friction force conversion. It can be seen from Table 3 that the degree of dispersion of SJ4 and SJ5 are larger, the coefficient of variation of SJ6 is 0.081, and the degree of dispersion is the smallest.

**Figure 11:**
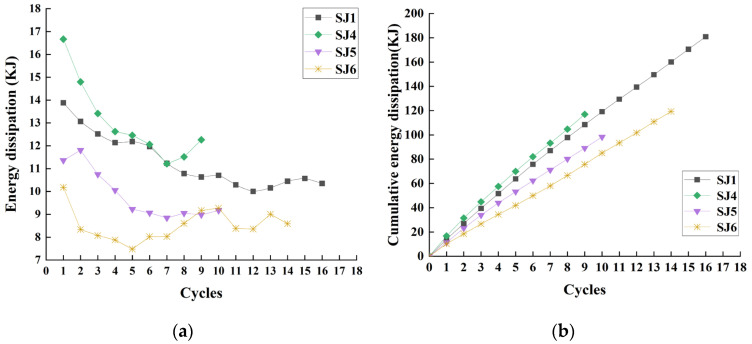
The energy dissipation analysis of SJ1, SJ4, SJ5, and SJ6: (**a**) Energy dissipation; (**b**) Cumulative energy dissipation.

In the case of different friction surfaces with the loading process, the energy dissipation of the specimens and their cumulative energy dissipation are shown in Figure 11. Although the energy dissipation capacity of SJ4 is better than other specimens, it decays faster. The decay rate of the energy dissipation capacity of SJ5 is between SJ1 and SJ4, the cumulative energy dissipation capacity is not as good as SJ1 and SJ4, and the energy dissipation capacity is not stable. The energy dissipation capacity of SJ6 decreases greatly during the second cyclic loading. As the loading progresses, due to the influence of debris, its energy dissipation capacity decreases first and then increases and finally tends to be stable.

### 3.3. Calculation of Ultimate Bearing Capacity

The ultimate bearing capacity of the bolt groups is the horizontal ultimate bearing capacity of the specimens. The design value of the bearing capacity of a single bolt should be the smaller of the shear capacity and the bearing capacity, and the bearing capacity of the bolt should be calculated according to the specification [24]:

(4) $$N_{v}^{b}=n_{v}\frac{\pi d^{2}}{4}f_{v}^{b}$$

(5) $$N_{c}^{b}=d\sum tf_{c}^{b}$$

In the formula, $n_{v}$ is the number of shear surfaces, d is the diameter of the bolt, ∑t is the smaller value of the total thickness of the bearing member, and $f_{v}^{b}$ and $f_{c}^{b}$ are the design values of the shear and compressive strength of the bolt, respectively. Since the bolt spacing, $l_{1}=550\;mm$, is greater than 15$d_{0}$ ($d_{0}$ is the aperture), the design value of the bearing capacity of the bolt should be multiplied by the reduction factor η:

(6) $$\eta =1.1-\frac{l_{1}}{150d_{0}}$$

It is calculated that the design value of the bearing capacity of a single M12 bolt in this paper should be a smaller value of $N_{c}^{b}=33.75\;\mathrm{kN}$. In the specimen with 16 bolts, the design value of the bearing capacity is calculated according to the bolt group formula to be 539.97 kN, SJ3 is loaded to 917 kN with a 100 t actuator, and the load is much larger than the calculated value of the specification; it is close to the ultimate loading value of the actuator, and the specimen is still not damaged. The calculated value of the specification is the design value of the bearing capacity that is biased towards safety, considering the reduction of many aspects that are smaller than the actual ultimate bearing capacity. Therefore, the ultimate bearing capacity of the composite chord calculated according to the specification is safe and feasible.

## 4. Finite Element Analysis

### 4.1. Finite Element Modeling

Finite element modeling for the friction energy dissipation composite chord specimens was performed using the finite element (FE) software ABAQUS. Solid elements (C3D8R) were employed for the specimens, the rigid-perfectly plastic model was used to fit the restoring force model of the specimens. Geometric nonlinearity was taken into account, with yield stress $f_{y}=345$ MPa, plastic strain 0, elastic modulus $E_{S}=2.06\times 10^{5}$ MPa, and Poisson’s ratio ν=0.3. The explicit solver in ABAQUS was used for analysis. Interlocking is random, so the influence of interlocking was not considered in the simulation. The finite element model for each specimen was established by using the Coulomb law of friction:

(7) $$\mu =\frac{F}{nF_{p}}$$

**Figure 12:**
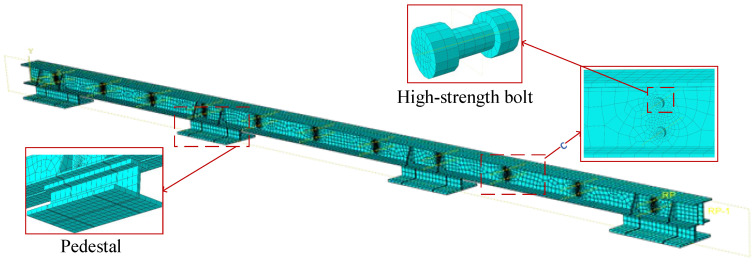
The finite element model of the friction energy dissipation composite chord.

In the formula, μ is the sliding friction coefficient, n represents the number of friction surfaces, and $F_{p}$ is the total clamping forces of bolts. Table 4 shows the average sliding friction coefficient of the test values. An interaction “surface to surface contact” was established between the friction contact parts. In the contact property setting, the normal behavior was set to “hard” contact, the “friction formulation” of the tangential behavior was set to “penalty” and input the friction coefficient in Table 4. In addition, the normal behavior was set to “hard” contact between the bolt rod and the slotted bolt holes, the bolt was bound to the angle steel by the “tie” command, and the “bolt load” was used to apply preload on the bolts. The T-section steel, bolts, and angle steel are partitioned to obtain the ideal mesh, the displacement load was applied to the T-section steel to simulate the load applied to the loading beam, and unnecessary degrees of freedom were fixed so the loading scheme was consistent with the test. The finite element model is shown in Figure 12.

### 4.2. Model Validation

**Figure 13:**
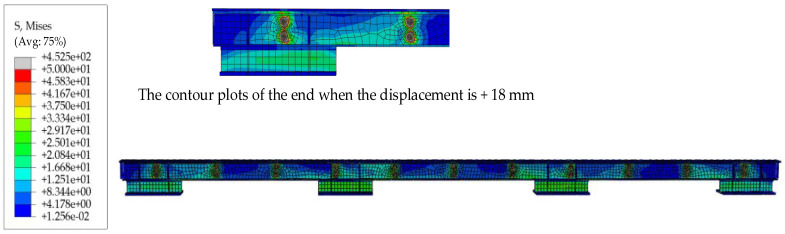
The contour plots of the finite element model results (based on SJ1).

**Figure 14:**
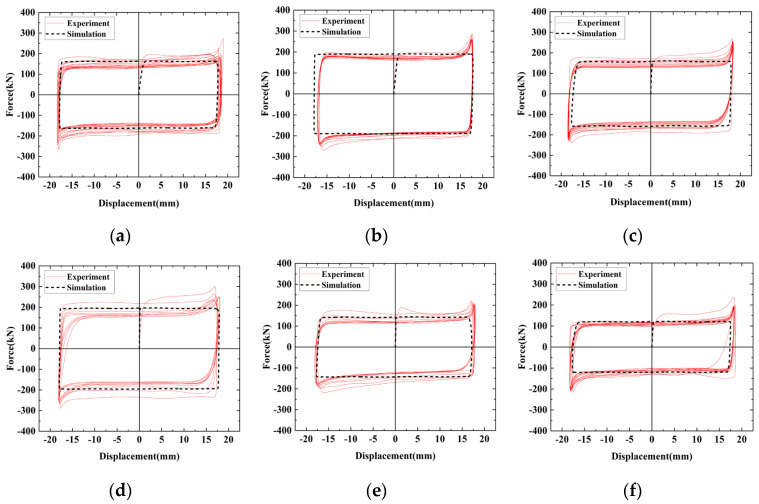
The comparison of hysteresis curves between the finite element simulation and the experiment: (**a**) SJ1; (**b**) SJ2; (**c**) SJ3; (**d**) SJ4; (**e**) SJ5; (**f**) SJ6.

Figure 13 shows the contour plots of the finite element model results, and Figure 14 compares the hysteresis curves of the FE model and the experiment at the same loading level. The hysteresis curves of the FE model and the test are in perfect agreement and are presented as an ideal rectangular hysteresis loop. There are no sharp parts at the edge of the hysteresis curves, and the sliding friction force does not fluctuate because the FE models ignore the damage to the friction surfaces and the extrusion deformation of the bolts during the sliding process; thus, the FE model is a more ideal state. Table 5 compares the average sliding friction force values for the test and FE results. The clamping forces of high-strength bolts are reduced to varying degrees in the test, while the clamping forces in the FE models are a fixed value, resulting in the finite element calculation result being slightly higher than the test value.

### 4.3. Effect of the Clamping Forces of Bolts on the Energy Dissipation Capacity of Chords

The energy dissipation capacity of the friction energy dissipation chord is related to the clamping forces of high-strength bolts. The design value, *P*, of the clamping force of a single high-strength bolt [24] is:

(8) $$P=\frac{0.9\times 0.9\times 0.9}{1.2}f_{u}A_{e}=0.6075f_{u}A_{e}$$

In the formula, 0.9 represents the reduction of the bolt material, the effect of super tension, and the additional safety factor; 1.2 is the minimum tensile strength of the bolt considering the adverse effect of shear stress; $f_{u}$ is the minimum tensile strength of the bolt, where the 10.9 grade bolt takes $f_{u}=1040\;N/mm^{2}$; $A_{e}$ is the effective cross-sectional area of the bolt, and the M12 high-strength bolt takes $A_{e}=84\;mm^{2}$. Therefore, the design value of the clamping force of a single high-strength bolt in this paper is P=53.07 kN, and the clamping force of a single M12 bolt cannot exceed 53.07 kN.

**Figure 15:**
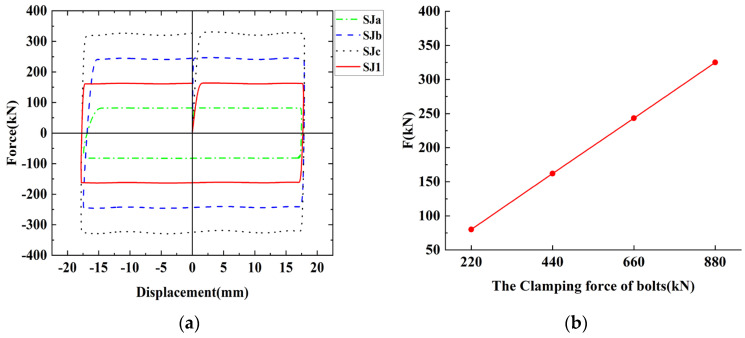
The comparison of hysteresis curves and average sliding friction force of the finite element simulation with different clamping force of bolts: (**a**) The comparison of hysteresis curves; (**b**) The comparison of the average sliding friction force.

Based on the finite element model of SJ1, in order to avoid sliding friction that was too great or too small, we kept the number of bolts unchanged at 22, and only the clamping force of the bolts was changed. Three FE models, Sja, SJb, and SJc, with the clamping force of a single M12 bolt of 10 kN, 30 kN, and 40 kN were established. Thus, Sja, SJb, and SJc, with a total clamping force of 220 kN, 660 kN, and 880 kN were compared with the finite element model of SJ1 to explore the effect of the clamping force of bolts on the energy dissipation capacity of chords. Table 6 provides a comparison of the friction force of the finite element models, and Figure 15 is a comparison of the hysteresis curves and the comparison of the average sliding friction force. In the FE simulation, as the clamping force increases, the dispersion degree of friction also increases. The average sliding friction is nearly proportional to the clamping force of the bolts, which conforms to the Coulomb law of friction because only the clamping force of the bolts is changed. Their sliding displacements are the same, the envelope area of the hysteresis curves is nearly proportional to the average sliding friction and, with the increase in clamping forces, the energy dissipation capacity of the chord increases. However, as the clamping force increases, the specimen is affected by interlocking and the degree of cyclic wear will be greater, resulting in faster friction attenuation. The finite element models are based on an ideal rigid plastic simulation, which can not simulate the friction wear behavior of the actual contact surfaces. Therefore, in practical engineering, in addition to selecting the appropriate clamping force of the bolts to control friction energy dissipation by using the Coulomb law of friction, interlocking and wear loss should be considered to avoid excessive clamping force, which may cause the friction of the chord to attenuate too fast and the sliding load to be too large, causing the component to fail to slide during buckling.

### 4.4. Effect of the Slotted Bolt Hole Length on the Energy Dissipation Capacity of Chords

**Figure 16:**
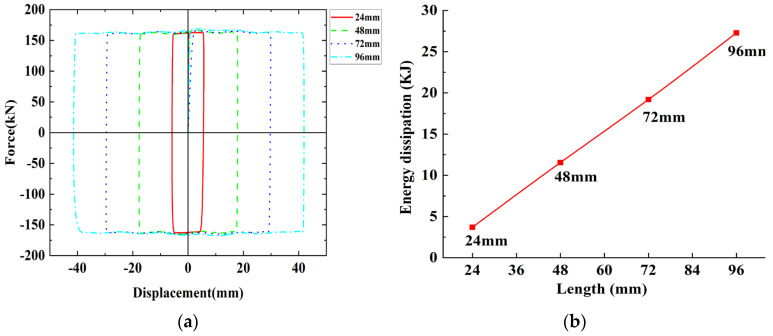
The comparison of hysteresis curves and energy dissipation of the finite element simulation with different slotted bolt holes length: (**a**) The comparison of hysteresis curves; (**b**) The comparison of energy dissipation.

Based on the FE model of SJ1, only the slotted bolt hole length was changed. Four FE models with slotted bolt hole lengths of 24 mm, 48 mm, 72 mm, and 96 mm were established and compared with the FE model of SJ1 to explore the influence of the slotted bolt hole lengths on the energy dissipation capacity of chords. Table 7 provides the comparison of the friction of the FE models, and Figure 16 is the hysteresis curve comparison of the FE models and the comparison of energy dissipation. There is no clear relationship between the sliding friction force and the slotted bolt hole lengths, but the envelope area of the hysteresis curves increases with the increase in length, and the energy dissipation capacity and the slotted bolt hole lengths are close to a linear relationship. Therefore, increasing the hole length within a certain limit can effectively enhance the energy dissipation capacity of the chord. The slotted bolt hole lengths can be set according to the requirements of the inter-story drifts of the truss. 

## 5. The Design Method of the Angle Steel

**Figure 17:**
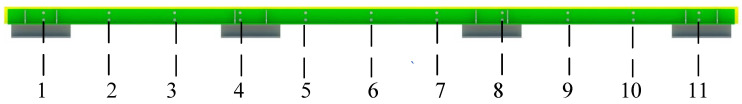
Area division of the angle steel of friction energy dissipation composite chord.

**Figure 18:**
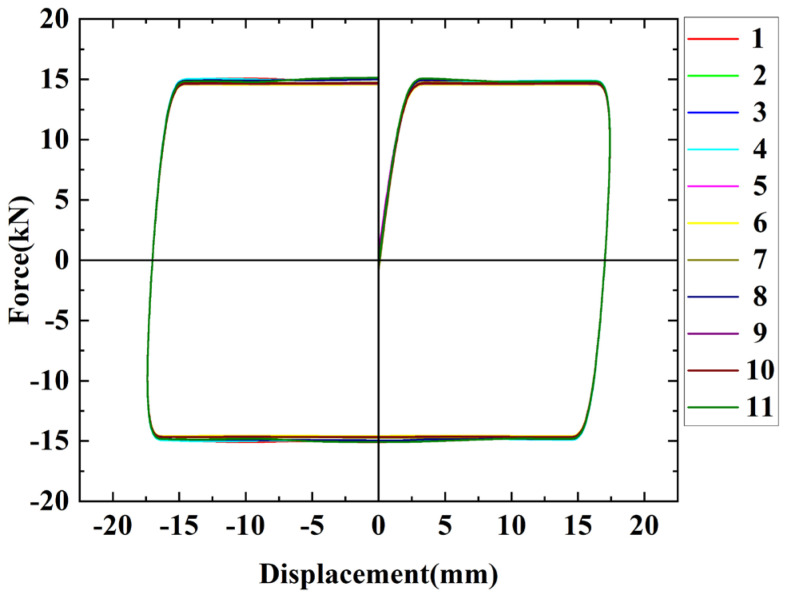
Relationship between friction and displacement in each area of the angle steel of friction energy dissipation composite chord.

Based on the FE model of SJb, the vicinity of each pair of high-strength bolts is numbered from No. 1~No. 11, as shown in Figure 17. The friction forces near No. 1~No. 11 bolts on the contact surface are extracted, and the relationship between friction and displacement in each area of angle steel is obtained, as shown in Figure 18. It can be seen that the friction in different areas of the angle steel is the same. In order to simplify the calculation, each bolt is set to provide friction f:

(9) $$f=\frac{\bar{F}}{n}$$

**Figure 19:**
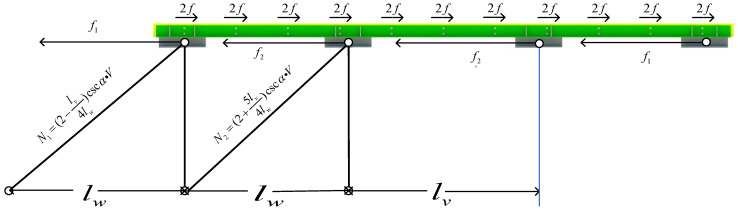
The axial force analysis of the web member of friction energy dissipation composite chord under horizontal force.

In the formula, n is the number of bolts and $\bar{F}$ is the average sliding friction force of the angle steel. The pedestal simulates the constraint effect of the truss web member on the friction energy dissipation composite chord. The calculation formula of the axial force, $N_{1}$ and $N_{2}$, of the web member under horizontal force is obtained according to the analysis of reference [28], as shown in Figure 19. α is the angle between the diagonal web member and the horizontal plane and V is the shear force of the upper and lower chords in the middle area; they are equivalent [29,30]. B1~B4 represents the pedestal, and $f_{1}$ and $f_{2}$ are the horizontal reaction forces provided by the pedestal, respectively:

(10) $$\frac{f_{1}}{f_{2}}=\frac{N_{1}}{N_{2}}=\frac{(2-\frac{l_{v}}{4l_{w}})\csc\alpha \times V}{(2+\frac{5l_{v}}{4l_{w}})\csc\alpha \times V}$$

(11) $$f_{1}+f_{2}=\frac{\bar{F}}{2}$$

**Figure 20:**
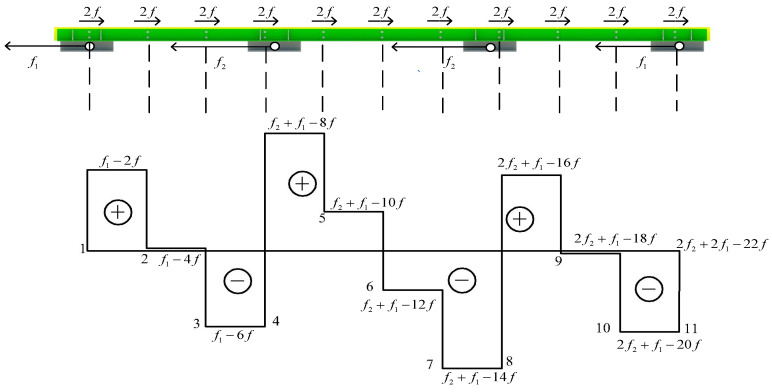
Axial force of the angle steel of friction energy dissipation composite chord.

The force analysis of the angle steel is carried out to obtain the axial force diagram of the angle steel, as shown in Figure 20.

In order to verify the accuracy of the calculation method in this paper, based on the FE model of SJb, *n* = 22, $\bar{F}=320.3\;kN$, $l_{w}=1200\;\mathrm{mm}$, and $l_{v}=1380\;\mathrm{mm}$, the axial force of the angle steel near the middle area is relatively the largest. The horizontal reaction force and the maximum axial force of the FE model are extracted and compared with the calculation results, as shown in Table 8. The calculation results are close to the FE simulation results; therefore, the calculation method proposed in this paper is suitable for the calculation of the friction energy dissipation chord. It can be seen from Figure 20 that the area where the axial force of the angle steel is relatively maximum, as long as the maximum part of the axial force meets the design requirements of the angle steel, the entire friction energy dissipation composite chord meets the design requirements. Therefore, in order to simplify the calculation, the angle steel can be an axial compression member to design by using the calculation method in this paper.

## 6. Conclusions

This paper aimed to investigate the effects of the number of bolts and the friction surface on the energy dissipation capacity of friction dissipation composite chords by a pseudo-static test of six friction dissipation composite chord specimens. An FE analysis with different clamping forces and slotted hole lengths was conducted, and the hysteretic behavior and energy dissipation capacity were discussed. Based on the results, the conclusions can be drawn as follows:

1. The hysteresis curve of the friction energy dissipation chord shows an approximately ideal rectangle, and the symmetry of the hysteresis curve is good. The energy dissipation capacity generally decreases with an increase in cyclic loading and then tends to be stable. The static and dynamic friction force conversion of the friction energy dissipation chord is relatively stable, and the friction plate can improve the stability of the static and dynamic friction force conversion.
2. With an increase in bolts, the energy dissipation capacity of the specimen decreases more slowly and cumulative energy dissipation is greater. It is suggested that bolt spacing can be considered to be between 3~23 times the bolt aperture to design the number. Friction energy dissipation chords with brass or aluminum friction plates show excellent energy dissipation capacity, but the energy dissipation capacity of the aluminum friction plate decays faster. Friction plates can improve the stability of hysteresis performance. The energy dissipation capacity of the specimen without a friction plate is not stable, and the energy dissipation capacity of the shot-blasting-treated specimen is greatly decayed after the first cycle. In practical engineering, it is recommended to use brass as the friction plate of the friction energy dissipation composite chord.
3. The ideal rigid plastic model is used to fit the hysteretic response model of friction energy dissipation chord. The model is simple and the fitting result is good. By analyzing the FE models with different clamping forces of bolts and slotted hole lengths, it is found that with an increase in clamping forces, the energy dissipation capacity of the chord increases but the stability decreases. In practical engineering, in addition to using the Coulomb law of friction to select an appropriate clamping force to control friction energy dissipation, interlocking and wear loss should be considered to avoid the friction of the chord attenuating too fast or the sliding load being too large, causing the component to fail to slide during buckling. It is found that increasing the hole length within the allowable range can effectively enhance the energy dissipation capacity of the chord, and its length can be set according to the demand of the inter-story drifts of the truss.
4. Under the horizontal force, the friction near each high-strength bolt is equal. Based on this finding, the force of angle steel is analyzed, and the design method of angle steel section is proposed.

## Figures and Tables

**Table 1:** The details of the friction energy dissipation composite chord specimens.

Specimens	Number of Bolts	Distance of Bolts/mm	Clamping Forces/kN	Friction Surface	Research Direction
SJ1	22	375	440	Brass	Comparative analysis of different numbers of bolts
SJ2	30	280	440	Brass	
SJ3	16	550	440	Brass	
SJ1	22	375	440	Brass	Comparative analysis of different arrangements of friction surfaces
SJ4	22	375	440	Aluminium	
SJ5	22	375	440	No friction plate	
SJ6	22	375	440	Shot blasting and without friction plate	

**Table 2:** The friction non-uniformity coefficient α and the influence coefficient of the maximum static friction force β.

Specimens	$F_{+}$ /kN	$F_{-}$ /kN	$F_{0+}$ /kN	$F_{0-}$ /kN	α	β
SJ1	153.3	−166.1	199.3	−178.0	0.92	1.18
SJ2	177.1	−197.8	193.5	−207.4	0.90	1.07
SJ3	150.5	−160.2	186.1	−182.7	0.94	1.19
SJ4	190.6	−194.1	230.0	−232.8	0.98	1.20
SJ5	136.5	−144.6	203.5	−189.1	0.94	1.39
SJ6	116.5	−121.0	159.4	−151.9	0.96	1.31

**Table 3:** Friction analysis of the friction energy dissipation composite chord specimens.

Specimens	${\bar{F}}_{\max}$ /kN	${\bar{F}}_{\min}$ /kN	${\bar{F}}_{\max}/{\bar{F}}_{\min}$	F	σ	C.V
SJ1	192.8	138.9	1.39	160.2	16.21	10.12%
SJ2	198.9	172.7	1.15	187.4	7.58	4.04%
SJ3	191.3	137.1	1.40	154.9	15.77	10.18%
SJ4	231.5	155.7	1.49	192.7	24.24	12.58%
SJ5	164.0	122.8	1.34	140.8	15.24	10.82%
SJ6	141.5	103.9	1.36	118.8	9.64	8.11%

**Table 4:** The average sliding friction coefficient of the friction energy dissipation composite chord specimens.

Specimens	SJ1	SJ2	SJ3	SJ4	SJ5	SJ6
μ	0.182	0.213	0.176	0.219	0.160	0.135

**Table 5:** The comparison of the average sliding friction force values between the finite element simulation and the experiment.

Specimens	Test/kN	Simulation/kN	Error
SJ1	159.9	162.1	1.4%
SJ2	187.6	188.4	0.4%
SJ3	155.2	157.2	1.3%
SJ4	192.3	186.3	3.1%
SJ5	140.4	142.2	1.3%
SJ6	118.9	119.4	0.4%

**Table 6:** The comparison of the friction force of the finite element simulation with different clamping force of bolts.

FE Model	Clamping Force/kN	Law/kN	Simulation/kN	Error	σ	C.V
SJ1	440	160.2	162.1	1.2%	0.84	0.52%
SJa	220	80.08	82.0	2.4%	0.26	0.32%
SJb	660	240.2	243.3	1.3%	1.81	0.74%
SJc	880	320.3	325.0	1.5%	2.73	0.84%

**Table 7:** The comparison of the friction force of the finite element simulation with different slotted bolt holes length.

L/mm	Law/kN	Simulation/kN	Error	σ	C.V
24	160.2	162.2	1.2%	0.54	0.0033
48	160.2	162.2	1.2%	4.11	0.0253
72	160.2	162.3	1.3%	4.46	0.0275
96	160.2	163.0	1.7%	4.26	0.0261

**Table 8:** The comparison of axial force between the simulation and the calculation results.

Force	Simulation/kN	Calculation/kN	Error
$f_{1}$	62.86	59.17	−5.87%
$f_{2}$	97.63	100.98	3.72%
No. 4~No. 5	42.96	43.67	1.63%
No. 7~No. 8	−43.85	−43.69	−0.37%

## Data Availability

No additional data is covered in this article.
